# Utilizing the New Glucometrics: A Practical Guide to Ambulatory Glucose Profile Interpretation

**DOI:** 10.17925/EE.2022.18.1.20

**Published:** 2022-06-13

**Authors:** John Doupis, Edward S Horton

**Affiliations:** 1. Department of Internal Medicine and Diabetes, Salamis Naval and Veterans Hospital, Salamis, Attiki, Greece; 2. Iatriko Paleou Falirou Medical Center, Diabetes Clinic, Athens, Greece; 3. Harvard Medical School, Boston, MA, USA

**Keywords:** Ambulatory glucose profile, continuous glucose monitoring, type 1 diabetes mellitus, type 2 diabetes mellitus, time in range, time below range, hypoglycaemia, glycaemic variability

## Abstract

Traditional continuous glucose monitoring and flash glucose monitoring systems are proven to lower glycated haemoglobin levels, decrease the time and impact of hypoglycaemia or hyperglycaemia and, consequently, improve the quality of life for children and adults with type 1 diabetes mellitus (T1DM) and adults with type 2 diabetes mellitus (T2DM). These glucose-sensing devices can generate large amounts of glucose data that can be used to define a detailed glycaemic profile for each user, which can be compared with targets for glucose control set by an International Consensus Panel of diabetes experts. Targets have been agreed upon for adults, children and adolescents with T1DM and adults with T2DM; separate targets have been agreed upon for older adults with diabetes, who are at higher risk of hypoglycaemia, and women with pregestational T1DM during pregnancy. Along with the objective measures and targets identified by the International Consensus Panel, the dense glucose data delivered by traditional continuous glucose monitoring and flash glucose monitoring systems is used to generate an ambulatory glucose profile, which summarizes the data in a visually impactful format that can be used to identify patterns and trends in daily glucose control, including those that raise clinical concerns. In this article, we provide a practical guide on how to interpret these new glucometrics using a straightforward algorithm, and clear visual examples that demystify the process of reviewing the glycaemic health of people with T1DM or T2DM such that forward-looking goals for diabetes management can be agreed.

For people with type 1 diabetes mellitus (T1DM) or type 2 diabetes mellitus (T2DM), glycaemic control has been monitored by two key measurements: laboratory-tested glycated haemoglobin (HbA1c) level and the individuals' self-monitored blood glucose (SMBG) fingerprick testing.^1,2^ Both of these measurements have significant limitations. HbA1c provides a surrogate assessment of the average glucose levels of the previous 3 months and produces no insight into the daily or weekly glucose fluctuations occuring during that period.^1,2^ Furthermore, HbA1c level is influenced by a range of non-glycaemic factors, which can make it an unreliable measure under different metabolic conditions such as anaemia, kidney disease or pregnancy.^1,2^ SMBG tests can provide an accurate measurement of capillary blood glucose at the moment of testing, but are limited by the practicalities of user technique and motivation to perform multiple daily tests because of the pain or discomfort associated with finger pricking, which may result in insufficient blood-glucose testing to achieve glucose control targets as indicated by guidelines. For example, 64% of people with diabetes fail to adhere to SMBG testing, as recommended by their healthcare professional (HCP).^3^ The significant limitations of this standard assessment of glucose control are overcome by the use of continuous glucose monitoring (CGM) for people with diabetes.

Traditional CGM and flash glucose monitoring (FLASH) systems measure glucose in the interstitial fluid rather than in the blood, and their use was proven to reduce the occurrence of clinically significant hypoglycaemia for people with T1DM and those with T2DM.^4–6^ Reduced rates of hypoglycaemia below 70 mg/dL and below 54 mg/dL are accompanied by reductions in hyperglycaemia; thus, using traditional CGM or FLASH is associated with lowered long-term HbA1c for people with T1DM or T2DM.^7–9^ In fact, evidence from real-world studies clearly indicate that the observed patterns of change in HbA1c do not differ between patients with T1DM and T2DM after they start using FLASH.^10^ It is evident that FLASH can be used in the same way in either adults with T1DM or those with T2DM to reduce long-term glucose exposure^,^ and experts support the use of CGM and FLASH systems to become a standard of care for people with T1DM and T2DM on basal-bolus therapy or on basal insulin alone.^10,11^ Importantly, FLASH is also associated with reduced acute diabetes events and incidence of hospital admissions for diabetic ketoacidosis and severe hypoglycaemia both in people with T1DM and in those with T2DM.^12^

Many of the day-to-day benefits of using CGM and FLASH systems can be related to the simple on-demand features that provide more frequent, painless insights into current glucose levels that accompany each scan of the glucose sensor, along with the glucose trend arrows that indicate the direction and rate of change in glucose levels.^13,14^ These allow users with T1DM or T2DM to understand their glucose fluctuations and make appropriate decisions on food consumption and insulin dosing, with consequent improvements in treatment satisfaction and reduced burden of diabetes. Real-world data from nearly 280,000 FLASH glucose sensors show that increased scanning behaviour is associated with improved measures of glycaemic control.^15^ Together, these benefits result in a significant improvement in quality of life for people living with T1DM or T2DM.^16^ The other significant benefit of using CGM or FLASH systems is the daily collection of hundreds of consecutive glucose readings that can be used to generate a dynamic picture of glucose control over days and weeks; this collection can be used to understand the patterns and trends in glucose levels for each person and how to optimize glycaemic control in line with improved diabetes health. Understanding of patterns and trends and how to optimize glycaemic control in line with improved diabetes health is achieved using the ambulatory glucose profile (AGP).^17,18^

In this practical clinical review, we look at the essential components of the AGP report format and how to use them in a patient-centred diabetes review. We provide insights into the objective and visual tools that summarize the considerable volume of glucose data that are collected by traditional CGM and FLASH systems. In doing so, we will focus on the report structures provided by the FreeStyle Libre system (Abbott Diabetes Care, Alameda, CA, USA). The FreeStyle Libre sensor has a 14-day wear time, during which it automatically takes and stores a glucose reading every minute. Glucose values are transmitted either to a dedicated reader or to the FreeStyle LibreLink smartphone app every time they are used to scan the sensor. Using either the reader or the FreeStyle LibreLink app also enables the user to record additional valuable information, such as mealtime carbohydrate intake, insulin doses and timings. In addition, the FreeStyle LibreLink app enables periods of physical activity or exercise to be recorded. These can all be helpful during an AGP review. Data collected by the FreeStyle LibreLink app are automatically uploaded to the cloud every time the phone is connected to the internet. Once uploaded, all glucose data are available to view in the LibreView digital diabetes system, from where glucose management reports are automatically collated to be examined by HCPs and by patients. The ability to access this glucose data remotely has created the reality of telemonitoring and telemedicine in diabetes care, allowing HCPs and people with diabetes to collaborate on diabetes management goals without needing to attend a physical diabetes centre.^19,20^ The efficacy of this technology has been most clearly validated during the COVID-19 pandemic, during which glycaemic control did not deteriorate amongst children and adults with diabetes using traditional CGM or FLASH systems, despite restricted in-person access to standard care, and even improved for the majority of users.^21^

## Components of the ambulatory glucose profile

The AGP is an internationally recognized standard for interpreting glucose control for people with diabetes using CGM or FLASH systems.^17,18,22,23^ It is an evolving tool for understanding the daily and weekly glycaemic challenges of living with diabetes and for shared decision-making and therapeutic adjustment.

### Data capture

The accurate interpretation of AGP data requires first the collection of sufficient glucose data on which to base confident decisions about the observed glucose trends. Studies have shown that 14 consecutive days of CGM or FLASH sensor use, with ≥70% data capture, is sufficient to generate an AGP report that will satisfactorily represent the patterns and trends in glycaemic control that support confident predictions of glucose exposure over 3 months.^24,25^

### Time in range

The availability of large amounts of glucose data for people using CGM or FLASH systems has led to setting standardized glucose target levels that a person with diabetes should be encouraged to achieve. These are defined as time in range (TIR), time below range (TBR) and time above range (TAR). These measurements, as well as being a clear target for people with T1DM or T2DM, have been agreed upon by an International Consensus Group (*Supplementary Table 1*).^26^ Separate targets have been recommended also for women with T1DM during pregnancy and for people who are at higher risk of hypoglycaemia because of age, duration of diabetes, duration of insulin therapy or impaired awareness of hypoglycaemia. The International Consensus Group has also emphasized the importance of setting individual goals when implementing these recommendations in clinical practice.

TIR indicates the amount of time during which glucose readings are within a target glucose range of 70–180 mg/dL (or 63–140 mg/dL during pregnancy). Furthermore, TBR indicates the amount of time spent below the target glucose range (<70 mg/dL, or <63 mg/dL during pregnancy), and TAR refers to the amount of time spent above the target range (>180 mg/dL, or >140 mg/dL during pregnancy). Within the AGP report, TBR and TAR are divided into low/very low and high/very high ranges, depending on the profile of the person with diabetes. TIR, TBR and TAR are understandable, uncomplicated and objective targets that provide a consistent focus for people with diabetes and their HCPs. More importantly, TIR, TBR and TAR are immediately responsive to changes in medication, diet and lifestyle during day-to-day diabetes management, in a way that is not possible with HbA1c.

The importance of TIR in diabetes care is underlined by recent studies that have demonstrated that TIR 70–180 mg/dL is inversely correlated with the prevalence of the complications of diabetes, including retinopathy, peripheral neuropathy and cardiovascular disease.^27–31^

### Visual components of the ambulatory glucose profile graphic

**Figure 1:**
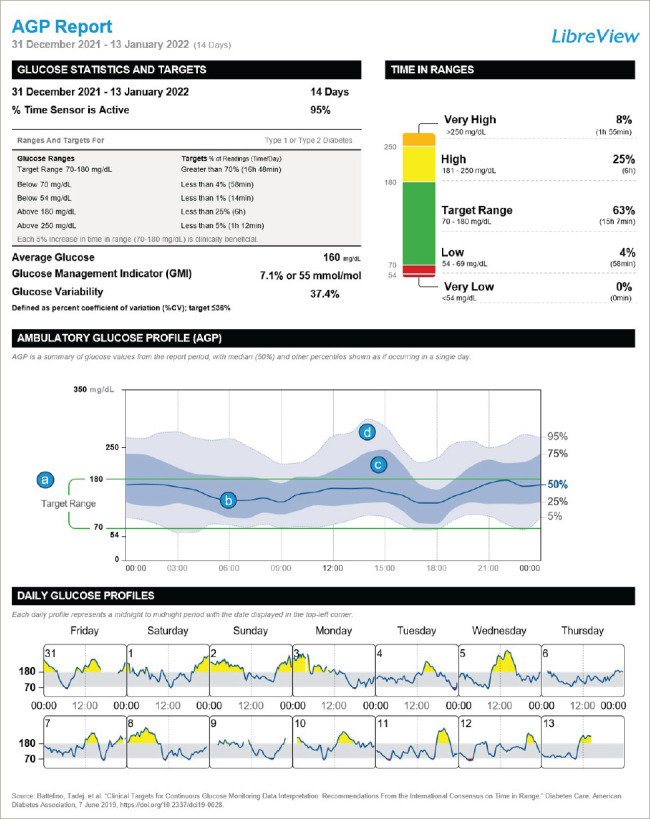
The key visual features of an ambulatory glucose profile report^26^*

The considerable amount of glucose data that is represented in a 14-day AGP is displayed as if all the readings had occurred in a single 24-hour period – the so-called ‘modal’ day.^17,18^ The visual elements of the AGP are constructed from four key features, as shown in *Figure 1* (labels a–d).^26^ (a) The target glucose range, which is shown as two green parallel lines, typically spans 70–180 mg/dL, except during pregnancy. (b) The median line is a dark blue line that traces the mid-point glucose reading as a measure of average glucose at each point in the modal day and shows whether the average glucose is within the target glucose range and how much it oscillates during the day. (c) The 25th–75th percentile band, also called the interquartile range (IQR), is a darker shaded band that shows the 50% of all glucose readings that are closest to the median line and their variability from day to day. The IQR band shows daily trends in glucose levels that happen on most days and indicates how medication and mealtimes are influencing glucose control. The times throughout the day when the IQR band is wider indicate more variability in glucose levels from day to day. (d) The 5th–95th percentile range, shown as a grey-shaded band, indicates the glucose readings that are less common. Glucose variability happens on some days but not others, and can indicate how behaviour and lifestyle issues impact glucose control. Notably, the 5% of glucose readings at the highest and lowest percentiles (i.e. those outside the 5th–95th percentile range) are not displayed in the AGP; as these values occur rarely, they would not affect clinical judgement and decision-making.

*The AGP Report is a standardized report developed by the International Diabetes Center. The example shown has been licenced and reproduced with permission from Abbott (Alameda, CA, USA) and downloaded from the LibreView diabetes data platform. The default setting is to show time in range for the target glucose range 70–180 mg/dL. To visualize metrics for a time in range of 63–140 mg/dL during pregnancy, the ‘Snapshot’ report is used.

**Figure 2:**
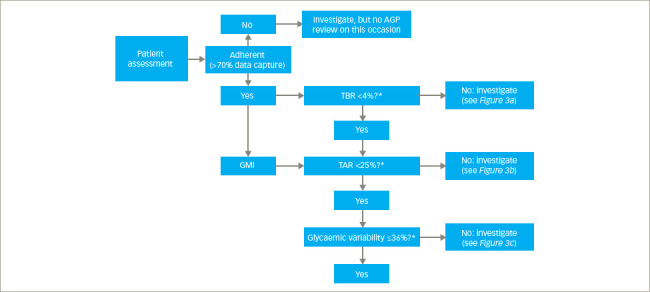
A systematic review of the ambulatory glucose profile report

*The targets for TBR, TAR and glycaemic variability are shown for people with T1DM or T2DM. Separate targets are defined for women withT1DM during pregnancy and for people who are at higher risk of hypoglycaemia because of age, duration of diabetes, duration of insulin therapy or impaired awareness of hypoglycaemia.

*AGP = ambulatory glucose profile; GMI = glucose management indicator; T1DM = type 1 diabetes mellitus; T2DM = type 2 diabetes mellitus; TAR = time above range; TBR = time below range.*

### Daily glucose profiles

At the very bottom of the AGP report are the daily glucose profiles, which show the glucose trace for each day of the 14-day AGP. By looking at these, it is possible to understand whether the glucose variability in the AGP can be interpreted in the context of different daily activities. For example, do weekdays and weekends show different glucose profiles that may contribute to the overall glucose control? Or are certain activities that happen on a regular day of the week worth investigating?

## Conducting a systematic ambulatory glucose profile review

Along with the visual elements of the AGP graph and the daily glucose profiles, the objective criteria provided by the TIR component of the AGP report, allow us to take a systematic approach in conducting a diabetes review and identifying aspects of glucose management that could be a focus for shared decisions on adjusting treatment or making changes to activities that are associated with glycaemic dysregulation. This is described in the algorithm presented in *Figure 2*.

### Step 1: Checking data capture

Before any productive review of glucose metrics generated by traditional CGM or FLASH systems can be conducted, the patient must have captured >70% of data over 14 consecutive days of sensor wear time. If their AGP report shows that the % Time Sensor is Active metric is >70%, the patient is considered to be adherent with using the system, and the review can move on to the next step. If the report shows that <70% of data has been captured, the patient is considered non-adherent with using the CGM or FLASH system, and further review using the AGP report should not be carried out. An insufficient use of the system may indicate the need for further education about system use or diabetes care, which is a separate objective.

### Step 2: Investigating time below range

Identifying hypoglycaemia is the priority of a systematic AGP review. Hypoglycaemia is the major limiting factor in the glycaemic management of T1DM or T2DM, and reducing both the occurrence and the risk of hypoglycaemia is at the heart of good diabetes care.^32^ If <4% of sensor glucose readings are below 70 mg/dL, the patient is on target, and the consultation can move on. If ≥4% of readings are below 70 mg/dL, it is important to understand why, especially if 1% or more are below 54 mg/dL; the international consensus recommends aiming for achieving <1% of readings below 70 mg/dL in high-risk individuals.

**Figure 3:**
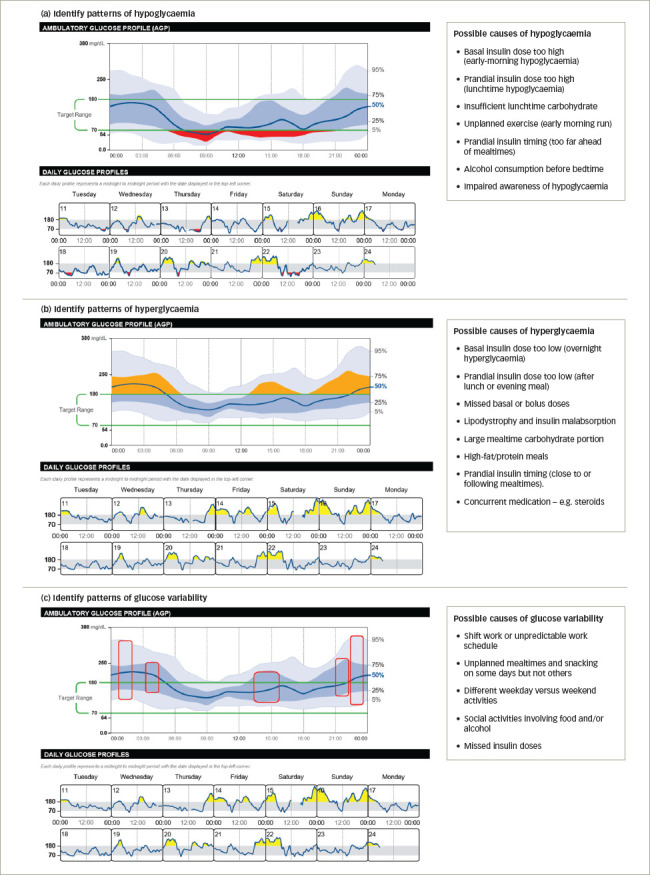
Identifying patterns in the ambulatory glucose profile

When evaluating patterns of hypoglycaemia (*Figure 3a*), the darker blue-shaded IQR band shows the glucose readings that are most consistent across each day. If this band approaches or dips below the target range, there is a regular trend of low glucose at these times of day, especially if the median line also dips into the hypoglycaemic zone. Where the lighter grey band strays below 70 mg/dL indicates occasional episodes of hypoglycaemia at these times; however, these are less predictable, as they do not reflect regular episodes of low glucose.

HCPs should talk to their patient and ask them about any activities or actions that may have contributed to their pattern of low glucose. They should explore whether an adjustment to daily treatment or daily activities at these times is needed. For example, is hypoglycaemia associated with fasting, exercise, alcohol consumption, stress or sickness, oral drug dosing, or overcorreaction to insulin? For less-predictable periods of low glucose, the patient may be able to pinpoint unplanned physical activities or unexpectedly missing mealtimes, for example.

Using the daily glucose profiles can provide further insights, as they provide a day-by-day log of the glucose readings for the days covered by the AGP. Examining individual days can confirm what was discussed with the patient during the investigation of the low glucose trends. The patient may also have logged details that are relevant to the discussion, including their carbohydrate consumption, insulin doses and timings, or periods of exercise, on their FreeStyle LibreLink app.

*Reproduced with permission from Abbott (Alameda, CA, USA).*

### Step 3: Investigating time above range

If <25% of sensor glucose readings are above 180 mg/dL, the patient is on target, and the consultation can move past this step. However, if ≥25% of readings are above 180 mg/dL, it is important to understand why, especially if 5% or more are above 250 mg/dL.

Consistent trends of high glucose are evident when the blue-shaded IQR band extends above the target range at these times of day (*Figure 3b*). You may also see the dark blue median line extend above the target range. These reflect regular trends of high glucose and are likely to be happening on most days. This is particularly important to note if the IQR band or the median line extend above 250 mg/dL.

HCPs should ask their patient about any activities or actions that may have contributed to their high glucose. These might include missed doses of oral medication or insulin injections and meals that may be larger than planned or contain a high fat and protein content. Does hyperglycaemia occur immediately after correcting for hypoglycaemia (i.e. is your patient reacting to low glucose)? The lighter grey band extending above 180 mg/dL indicates less-predictable episodes of hyperglycaemia at these times; however, they may be worth investigating, as any reduction in TAR will tend to increase TIR.

The daily glucose profiles can be used to help investigate the causes of high glucose; for example, diet and daily activities may differ significantly between weekdays and weekends (*Figure 3b*). Again, the patient may also have logged details that are relevant and informative to this discussion on their FreeStyle LibreLink app.

### Step 4: Investigating glucose variability

Evidence indicating an association between glucose variability and an increased risk of microvascular and macrovascular complications of diabetes has been emerging.^33–37^ Glucose variability is defined within the AGP report using the coefficient of variation of the standard deviation of mean glucose values (CV) and summarizes the glucose variability at a specific time between different days.^38^ The target for CV is ≤36%, as the risk of hypoglycaemic events rises significantly above this value.^39^ The percentage of CV (%CV) is considered a reliable marker for assessing the amplitude of glucose variation, as it is adjusted for the mean glucose value and is better correlated with TBR.^38^ However, alongside the %CV within any individual AGP reporting period, the stability of mean glucose from one review to the next can also be checked to assess glucose variability.

When investigating a CV of >36%, HCPs should look for areas of the AGP with a wider dark blue IQR band and a wider outer 5–95th percentile band (*Figure 3c*). Possible causes of glucose variability in the patient should be identified, focusing on what may be changing from one day to the next. A wider, dark blue IQR band indicates unwanted glucose fluctuations on most days, potentially signifying a need to adjust therapeutic parameters, such as medication doses or timings, or mealtime portion control. Where the lighter-shaded band is billowing, glucose variability is caused by occasional factors, such as unplanned meals and snacks, or intermittent exercise or routines during the weekday compared with the weekends. These suggested reasons for glucose variability can be confirmed using the daily glucose profiles, which help to visualize the times between individual days that glucose levels are consistently different.

### Step 5: The importance of the glucose management indicator in therapy adjustment

Following a review of hyperglycaemia or glucose variability, treatment intensification may be indicated to help patients make progress towards meeting consensus targets for TIR. This must involve an assessment of the glucose management indicator (GMI), alongside the most recent HbA1c value for a person with diabetes. GMI is a measure of short-term glucose exposure over the 14-day AGP assessment period, calculated from CGM-derived mean glucose.^40^ Importantly, it is reported using the same Diabetes Control and Complications Trial or International Federation of Clinical Chemistry units as HbA1c (% or mmol/mol). Although HbA1c is a surrogate for long-term glycaemic exposure, it is also influenced by a range of non-glycaemic factors; therefore, HbA1c may not reflect average glucose alone.^41^ As the GMI value is a true reflection of average glucose, it can help to guide treatment intensification by comparing it with the most recent HbA1c test result, and it also more precisely reflects the reality of mean glucose exposure, even when HbA1c varies from one visit to the next. If the GMI metric is higher than or comparable to the measured HbA1c, the patient can be considered a low or average glycator. In these cases, treatment intensification can be guided by GMI or HbA1c. However, if the GMI measure is demonstrably lower than a recent HbA1c, the patient is considered to be a high glycator, and treatment intensification should be managed using the GMI value. Treatment intensification based solely on HbA1c carries a risk of hypoglycaemia for high glycators.^41^ This assessment is important, given that the relationship between average glucose as measured by GMI and by HbA1c can differ based on many factors, including ethnic and racial differences.^42–44^

## Conclusions

In this guide to understanding and interpreting the AGP report format, we have provided practical insights into the diabetes management of people with diabetes in the context of traditional CGM and FLASH technologies. These outline the derivation and application of international consensus recommendations and targets for TIR, TBR and TAR. Furthermore, the value of using %CV as a measure of glycaemic variability and the importance of GMI in making therapeutic adjustments was emphasized. A productive diabetes review, involving shared decision-making, can be conducted with the patient by following a straightforward algorithm and considering each of the measures of diabetes health that are provided by the wealth of glucose data collected using CGM and FLASH systems. In 2022, this may be done in person or in a remote consultation. Although the importance of individualized, patient-centred care must always be emphasized, the objective and visual elements of the AGP report format provide a strong framework that supports the management of long-term glycaemic control for people with diabetes.
